# Ethyl 3-(2,4-dichloro­benzyl­idene)carbazate

**DOI:** 10.1107/S1600536809044420

**Published:** 2009-10-31

**Authors:** Yu-Feng Li, Hai-Xing Liu, Fang-Fang Jian

**Affiliations:** a Microscale Science Institute, Department of Chemistry and Chemical Engineering, Weifang University, Weifang 261061, People’s Republic of China; b Microscale Science Institute, Weifang University, Weifang 261061, People’s Republic of China

## Abstract

The title compound, C_10_H_10_Cl_2_N_2_O_2_, was prepared by the reaction of ethyl carbazate and 2,4-dichloro­benzaldehyde. In the crystal structure, mol­ecules are linked by inter­molecular N—H⋯O hydrogen bonds, forming extended chains along [001].

## Related literature

For applications of Schiff base compounds, see: Cimerman *et al.* (1997 ▶). For the C=N double-bond length in a related structure, see: Girgis (2006 ▶).
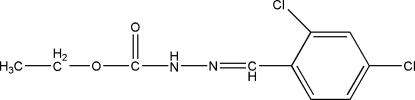


## Experimental

### 

#### Crystal data

C_10_H_10_Cl_2_N_2_O_2_
*M* *_r_* = 261.10
Tetragonal, 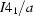

*a* = 18.021 (3) Å
*c* = 14.983 (3) Å
*V* = 4865.8 (14) Å^3^
*Z* = 16
Mo *K*α radiation
μ = 0.52 mm^−1^
*T* = 293 K
0.25 × 0.20 × 0.18 mm

#### Data collection

Bruker SMART CCD diffractometer
Absorption correction: multi-scan (*SADABS*; Sheldrick, 1996 ▶) *T*_min_ = 0.492, *T*_max_ = 0.729
21376 measured reflections
2789 independent reflections
2409 reflections with *I* > 2σ(*I*)
*R*_int_ = 0.045

#### Refinement

*R*[*F* ^2^ > 2σ(*F* ^2^)] = 0.046
*wR*(*F* ^2^) = 0.121
*S* = 1.09
2789 reflections
146 parameters
H-atom parameters constrained
Δρ_max_ = 0.37 e Å^−3^
Δρ_min_ = −0.50 e Å^−3^

### 

Data collection: *SMART* (Bruker, 1997 ▶); cell refinement: *SAINT* (Bruker, 1997 ▶); data reduction: *SAINT*; program(s) used to solve structure: *SHELXS97* (Sheldrick, 2008 ▶); program(s) used to refine structure: *SHELXL97* (Sheldrick, 2008 ▶); molecular graphics: *SHELXTL* (Sheldrick, 2008 ▶); software used to prepare material for publication: *SHELXTL*.

## Supplementary Material

Crystal structure: contains datablocks global, I. DOI: 10.1107/S1600536809044420/lh2929sup1.cif

Structure factors: contains datablocks I. DOI: 10.1107/S1600536809044420/lh2929Isup2.hkl

Additional supplementary materials:  crystallographic information; 3D view; checkCIF report

## Figures and Tables

**Table 1:** Hydrogen-bond geometry (Å, °)

*D*—H⋯*A*	*D*—H	H⋯*A*	*D*⋯*A*	*D*—H⋯*A*
N1—H1*A*⋯O1^i^	0.86	2.12	2.927 (2)	156

Symmetry code: (i) 
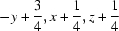
.

**Fig. 1.**
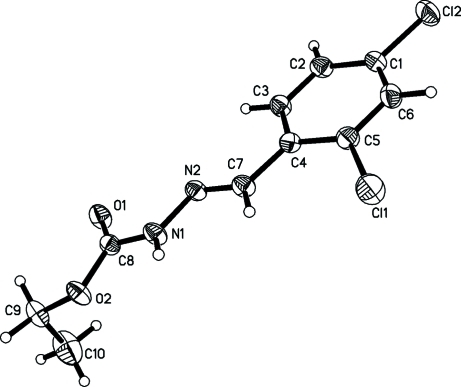
The molecular structure of the title compound showing 30% probability displacement ellipsoids and the atom-numbering scheme.

## supplementary crystallographic information

### Comment

Schiff bases have received considerable attention in the literature and have potential analytical applications (Cimerman *et al.*, 1997). As part of our search for new schiff base compounds we synthesized the title compound (I), and its crystal structure is determined herein.

The molcular structure of (I) is shown in Fig. 1. The C8—N1 bond length of 1.271 (2)Å is comparable with C—N double bond [1.281 (2) Å] reported in a related structure (Girgis, 2006). In the crystal structure, molecules are linked by intermolecular N-H···O hydrogen bonds to form extended chains along [001].

### Experimental

A mixture of the 2,4-dichlorobenzaldehyde (0.1 mol), and Ethyl carbazate (0.1 mol) was stirred in refluxing ethanol (20 mL) for 4 h to afford the title compound (0.080 mol, yield 80%). Single crystals suitable for X-ray measurements were obtained by recrystallization of a solution of (I) in ethanol at room temperature.

### Refinement

H atoms were fixed geometrically and allowed to ride on their attached atoms, with C—H distances = 0.93-0.97 Å; N-H = 0.86Å and with *U*_iso_(H) = 1.2*U*_eq_(C,N) or 1.5*U*_eq_(C_methyl_).

### Crystal data

**Table:**

C_10_H_10_Cl_2_N_2_O_2_	*D*_x_ = 1.426 Mg m^−^^3^
*M**_r_* = 261.10	Mo *K*α radiation, λ = 0.71073 Å
Tetragonal, *I*4_1_/*a*	Cell parameters from 1977 reflections
Hall symbol: -I 4ad	θ = 3.5–27.4°
*a* = 18.021 (3) Å	µ = 0.52 mm^−^^1^
*c* = 14.983 (3) Å	*T* = 293 K
*V* = 4865.8 (14) Å^3^	Block, colorless
*Z* = 16	0.25 × 0.20 × 0.18 mm
*F*(000) = 2144	

### Data collection

**Table:**

Bruker SMART CCD diffractometer	2789 independent reflections
Radiation source: fine-focus sealed tube	2409 reflections with *I* > 2σ(*I*)
graphite	*R*_int_ = 0.045
φ and ω scans	θ_max_ = 27.5°, θ_min_ = 3.2°
Absorption correction: multi-scan (*SADABS*; Sheldrick, 1996)	*h* = −23→23
*T*_min_ = 0.492, *T*_max_ = 0.729	*k* = −23→23
21376 measured reflections	*l* = −19→18

### Refinement

**Table:**

Refinement on *F*^2^	Secondary atom site location: difference Fourier map
Least-squares matrix: full	Hydrogen site location: inferred from neighbouring sites
*R*[*F*^2^ > 2σ(*F*^2^)] = 0.046	H-atom parameters constrained
*wR*(*F*^2^) = 0.121	*w* = 1/[σ^2^(*F*_o_^2^) + (0.0645*P*)^2^ + 2.5907*P*] where *P* = (*F*_o_^2^ + 2*F*_c_^2^)/3
*S* = 1.09	(Δ/σ)_max_ = 0.001
2789 reflections	Δρ_max_ = 0.37 e Å^−^^3^
146 parameters	Δρ_min_ = −0.49 e Å^−^^3^
0 restraints	Extinction correction: *SHELXL97* (Sheldrick, 2008), Fc^*^=kFc[1+0.001xFc^2^λ^3^/sin(2θ)]^-1/4^
Primary atom site location: structure-invariant direct methods	Extinction coefficient: 0.0030 (4)

### Special details

**Table:**

Geometry. All e.s.d.'s (except the e.s.d. in the dihedral angle between two l.s. planes) are estimated using the full covariance matrix. The cell e.s.d.'s are taken into account individually in the estimation of e.s.d.'s in distances, angles and torsion angles; correlations between e.s.d.'s in cell parameters are only used when they are defined by crystal symmetry. An approximate (isotropic) treatment of cell e.s.d.'s is used for estimating e.s.d.'s involving l.s. planes.
Refinement. Refinement of *F*^2^ against ALL reflections. The weighted *R*-factor *wR* and goodness of fit *S* are based on *F*^2^, conventional *R*-factors *R* are based on *F*, with *F* set to zero for negative *F*^2^. The threshold expression of *F*^2^ > σ(*F*^2^) is used only for calculating *R*-factors(gt) *etc*. and is not relevant to the choice of reflections for refinement. *R*-factors based on *F*^2^ are statistically about twice as large as those based on *F*, and *R*- factors based on ALL data will be even larger.

### Fractional atomic coordinates and isotropic or equivalent isotropic displacement parameters (Å^2^)

**Table:**

	*x*	*y*	*z*	*U*_iso_*/*U*_eq_	
Cl1	0.22311 (4)	0.76326 (3)	0.01502 (4)	0.0730 (2)	
Cl2	0.09897 (3)	0.79872 (3)	−0.30581 (4)	0.0661 (2)	
N2	0.28329 (7)	0.54137 (8)	−0.06890 (9)	0.0376 (3)	
O2	0.39424 (7)	0.41332 (7)	0.04214 (8)	0.0474 (3)	
N1	0.32058 (8)	0.50259 (8)	−0.00379 (9)	0.0416 (3)	
H1A	0.3228	0.5189	0.0501	0.050*	
O1	0.34815 (8)	0.40718 (7)	−0.09813 (8)	0.0500 (3)	
C8	0.35371 (9)	0.43809 (9)	−0.02677 (10)	0.0362 (3)	
C7	0.26094 (9)	0.60548 (10)	−0.04588 (11)	0.0411 (4)	
H7A	0.2695	0.6224	0.0119	0.049*	
C4	0.22188 (8)	0.65273 (9)	−0.10968 (10)	0.0371 (3)	
C2	0.16741 (10)	0.67119 (10)	−0.25620 (11)	0.0440 (4)	
H2B	0.1568	0.6534	−0.3130	0.053*	
C5	0.20078 (10)	0.72492 (10)	−0.08829 (11)	0.0441 (4)	
C3	0.20456 (10)	0.62758 (9)	−0.19537 (11)	0.0414 (4)	
H3A	0.2186	0.5798	−0.2119	0.050*	
C1	0.14623 (10)	0.74200 (10)	−0.23128 (12)	0.0445 (4)	
C6	0.16227 (11)	0.76940 (10)	−0.14764 (13)	0.0498 (4)	
H6A	0.1475	0.8170	−0.1314	0.060*	
C9	0.44238 (11)	0.35079 (11)	0.02348 (14)	0.0538 (5)	
H9A	0.4173	0.3167	−0.0166	0.065*	
H9B	0.4529	0.3246	0.0786	0.065*	
C10	0.51290 (15)	0.37543 (18)	−0.0177 (2)	0.0908 (9)	
H10A	0.5436	0.3330	−0.0297	0.136*	
H10B	0.5383	0.4083	0.0225	0.136*	
H10C	0.5025	0.4010	−0.0725	0.136*	

### Atomic displacement parameters (Å^2^)

**Table:**

	*U*^11^	*U*^22^	*U*^33^	*U*^12^	*U*^13^	*U*^23^
Cl1	0.1010 (5)	0.0595 (3)	0.0586 (3)	0.0176 (3)	−0.0303 (3)	−0.0252 (2)
Cl2	0.0726 (4)	0.0565 (3)	0.0693 (4)	0.0135 (2)	−0.0261 (3)	0.0084 (2)
N2	0.0396 (7)	0.0420 (7)	0.0312 (6)	0.0035 (6)	−0.0042 (5)	0.0013 (5)
O2	0.0540 (7)	0.0566 (7)	0.0316 (6)	0.0170 (6)	−0.0047 (5)	0.0045 (5)
N1	0.0505 (8)	0.0476 (8)	0.0266 (6)	0.0112 (6)	−0.0060 (5)	−0.0019 (5)
O1	0.0713 (8)	0.0455 (6)	0.0332 (6)	0.0116 (6)	−0.0084 (6)	−0.0044 (5)
C8	0.0401 (8)	0.0411 (8)	0.0275 (7)	0.0010 (6)	−0.0002 (6)	0.0048 (6)
C7	0.0445 (8)	0.0457 (9)	0.0332 (7)	0.0056 (7)	−0.0035 (6)	−0.0033 (7)
C4	0.0351 (7)	0.0399 (8)	0.0361 (8)	0.0016 (6)	−0.0014 (6)	−0.0019 (6)
C2	0.0458 (9)	0.0494 (9)	0.0367 (8)	0.0031 (7)	−0.0066 (7)	−0.0039 (7)
C5	0.0477 (9)	0.0423 (8)	0.0423 (9)	0.0029 (7)	−0.0076 (7)	−0.0086 (7)
C3	0.0451 (9)	0.0403 (8)	0.0388 (8)	0.0063 (7)	−0.0026 (7)	−0.0048 (7)
C1	0.0418 (9)	0.0437 (9)	0.0481 (9)	0.0038 (7)	−0.0090 (7)	0.0051 (7)
C6	0.0548 (10)	0.0383 (8)	0.0562 (11)	0.0077 (7)	−0.0096 (9)	−0.0067 (8)
C9	0.0595 (11)	0.0533 (10)	0.0484 (10)	0.0185 (9)	0.0017 (9)	0.0126 (8)
C10	0.0688 (15)	0.106 (2)	0.098 (2)	0.0277 (14)	0.0305 (14)	0.0353 (17)

### Geometric parameters (Å, °)

**Table:**

Cl1—C5	1.7421 (17)	C2—C3	1.377 (2)
Cl2—C1	1.7370 (17)	C2—C1	1.383 (2)
N2—C7	1.271 (2)	C2—H2B	0.9300
N2—N1	1.3755 (18)	C5—C6	1.384 (2)
O2—C8	1.3412 (19)	C3—H3A	0.9300
O2—C9	1.449 (2)	C1—C6	1.378 (3)
N1—C8	1.351 (2)	C6—H6A	0.9300
N1—H1A	0.8600	C9—C10	1.481 (3)
O1—C8	1.2097 (19)	C9—H9A	0.9700
C7—C4	1.461 (2)	C9—H9B	0.9700
C7—H7A	0.9300	C10—H10A	0.9600
C4—C5	1.393 (2)	C10—H10B	0.9600
C4—C3	1.397 (2)	C10—H10C	0.9600
			
C7—N2—N1	115.10 (13)	C2—C3—H3A	118.9
C8—O2—C9	115.87 (14)	C4—C3—H3A	118.9
C8—N1—N2	118.18 (13)	C6—C1—C2	121.22 (16)
C8—N1—H1A	120.9	C6—C1—Cl2	118.48 (13)
N2—N1—H1A	120.9	C2—C1—Cl2	120.30 (14)
O1—C8—O2	124.90 (15)	C1—C6—C5	118.83 (16)
O1—C8—N1	125.78 (15)	C1—C6—H6A	120.6
O2—C8—N1	109.31 (13)	C5—C6—H6A	120.6
N2—C7—C4	120.32 (14)	O2—C9—C10	111.15 (19)
N2—C7—H7A	119.8	O2—C9—H9A	109.4
C4—C7—H7A	119.8	C10—C9—H9A	109.4
C5—C4—C3	116.98 (15)	O2—C9—H9B	109.4
C5—C4—C7	121.69 (14)	C10—C9—H9B	109.4
C3—C4—C7	121.33 (14)	H9A—C9—H9B	108.0
C3—C2—C1	118.81 (16)	C9—C10—H10A	109.5
C3—C2—H2B	120.6	C9—C10—H10B	109.5
C1—C2—H2B	120.6	H10A—C10—H10B	109.5
C6—C5—C4	122.01 (15)	C9—C10—H10C	109.5
C6—C5—Cl1	117.20 (13)	H10A—C10—H10C	109.5
C4—C5—Cl1	120.79 (13)	H10B—C10—H10C	109.5
C2—C3—C4	122.12 (15)		

### Hydrogen-bond geometry (Å, °)

**Table:**

*D*—H···*A*	*D*—H	H···*A*	*D*···*A*	*D*—H···*A*
N1—H1A···O1^i^	0.86	2.12	2.927 (2)	156

Symmetry codes: (i) −*y*+3/4, *x*+1/4, *z*+1/4.
